# AQP4-dependent glioma cell features affect the phenotype of surrounding cells via extracellular vesicles

**DOI:** 10.1186/s13578-022-00888-2

**Published:** 2022-09-07

**Authors:** Laura Simone, Francesco Pisani, Elena Binda, Antonio Frigeri, Angelo L. Vescovi, Maria Svelto, Grazia P. Nicchia

**Affiliations:** 1 Cancer Stem Cells Unit, Fondazione IRCCS Casa Sollievo della Sofferenza, Viale Cappuccini, snc, 71013 San Giovanni Rotondo, Italy; 2 Department of Bioscience, Biotechnology and Biopharmaceutics, University of Bari Aldo Moro, Bari, Italy; 3 Center for Synaptic Neuroscience and Technology, Istituto Italiano di Tecnologia (IIT), Genoa, Italy; 4 School of Medicine, Department of Basic Medical Sciences, Neuroscience and Sense Organs, University of Bari Aldo Moro, Bari, Italy; 5 Department of Neuroscience, Albert Einstein College of Medicine, Yeshiva University, Bronx, New York, USA; 6 Istituto di Tecnologie Biomediche, Bari, Italy; 7 Institute of Biomembranes and Bioenergetics, National Research Council, Bari, Italy; 8 National Institute of Biostructures and Biosystems (INBB), Rome, Italy; 9 Consiglio Nazionale delle Ricerche (CNR), Istituto per la Sintesi Organica e la Fotoreattività (ISOF), Bologna, Italy

**Keywords:** GBM, EVs, Tumour environment, Apoptosis, Migration, AQP4

## Abstract

**Background:**

Extracellular vesicles (EVs) are membrane-enclosed particles released systemically by all cells, including tumours. Tumour EVs have been shown to manipulate their local environments as well as distal targets to sustain the tumour in a variety of tumours, including glioblastoma (GBM).

We have previously demonstrated the dual role of the glial water channel aquaporin-4 (AQP4) protein in glioma progression or suppression depending on its aggregation state. However, its possible role in communication mechanisms in the microenvironment of malignant gliomas remains to be unveiled.

**Results:**

Here we show that in GBM cells AQP4 is released via EVs that are able to affect the GBM microenvironment. To explore this role, EVs derived from invasive GBM cells expressing AQP4-tetramers or apoptotic GBM cells expressing orthogonal arrays of particles (AQP4-OAPs) were isolated, using a differential ultracentrifugation method, and were added to pre-seeded GBM cells. Confocal microscopy analysis was used to visualize the interaction and uptake of AQP4-containing EVs by recipient cells. Chemoinvasion and Caspase3/7 activation assay, performed on recipient cells after EVs uptake, revealed that EVs produced by AQP4-tetramers expressing cells were able to drive surrounding tumour cells toward the migratory phenotype, whereas EVs produced by AQP4-OAPs expressing cells drive them toward the apoptosis pathway.

**Conclusion:**

This study demonstrates that the different GBM cell phenotypes can be transferred by AQP4-containing EVs able to influence tumour cell fate toward invasiveness or apoptosis.

This study opens a new perspective on the role of AQP4 in the brain tumour microenvironment associated with the EV-dependent communication mechanism.

**Graphical Abstract:**

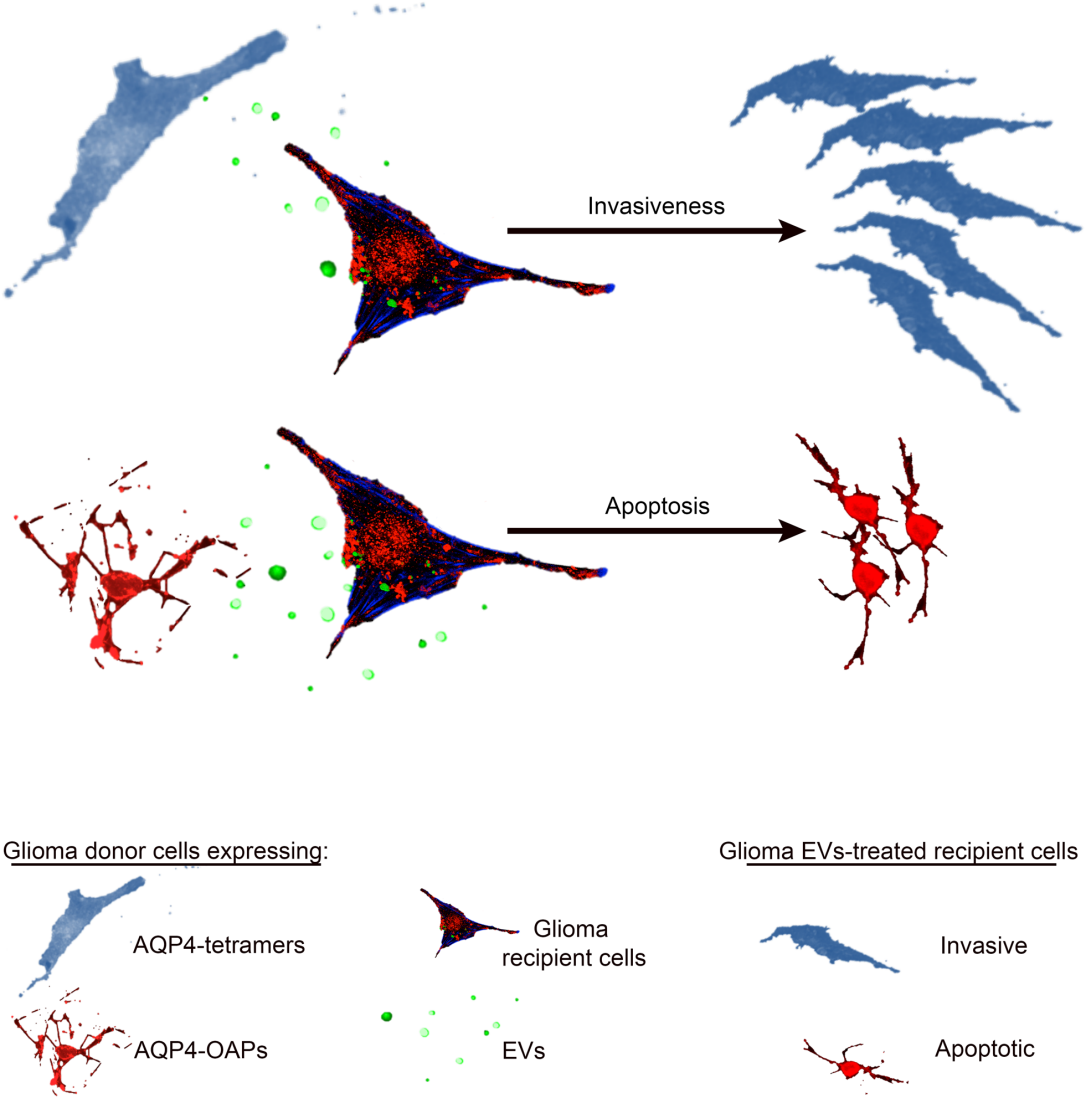

**Supplementary Information:**

The online version contains supplementary material available at 10.1186/s13578-022-00888-2.

## Background

Glioblastoma (GBM) is the most aggressive among tumours of glial origin and is characterized by cellular heterogeneity, rapid proliferation, angiogenesis, extensive invasion and a harsh intratumour microenvironment [1]. Besides tumour cells, the GBM tumour microenvironment (TME) also consists of a subpopulation of non-neoplastic cells, comprising astrocytes, vascular cells, stem-like glioma cells, peripheral immune cells, all deeply intermingled throughout the tumour mass [2]. It has been extensively demonstrated that GBM cells recruit normal cells in their environs to promote growth, sustenance and infiltration of the tumour into the brain. In addition to various tumour-stroma interactions, tumour cells have many interactions with surrounding malignant cells that are also essential to tumour growth and metastatic spread. Cell–cell communication occurs via the secretion and uptake of a number of factors that play a pivotal role in controlling the course of the disease, including signalling molecules able to bind membrane receptors to target cells, soluble factors and metabolites. However, the importance of other routes of communication, such as gap junctions and extracellular vesicles (EVs), are now being recognized [3].

EVs are a class of small bilayered particles that have the ability to transfer their molecular cargoes consisting of non secretable proteins, lipids, nucleic acids and even whole organelles to target cells, both locally and at a distance [4]. EVs are markedly heterogeneous in size, content and function [5]. The commonly studied subfractions of EVs are the large vesicles such as apoptotic bodies, oncosomes and small vesicles derived from cytoplasmic blebs that bud from the cell; the smallest EVs include exosomes that are formed by multivesicular bodies that fuse with the plasma membrane to exit the cell [6]. After release, EVs can be taken up by near or distant cells or interact with receptors of the recipient cell plasma membrane leading to direct or indirect stimulation of intracellular signalling cascades [7]. Functionally, in the context of cancer and in GBM, EVs cargos have been shown to be able to affect the phenotype of surrounding cells to sustain tumour growth and persistence [8].

The glial membrane water channel AQP4 holds pathological implications in the brain tumour context as it is involved in tumour-associated oedema, tumour cell invasion and proliferation [9]. AQP4 is expressed as different isoforms with different combinations of N-terminus and C-terminus [10]. Based on differences at the N-terminus, the two main isoforms are: M23-AQP4, able to aggregate into square well-ordered structures called orthogonal arrays of particles (OAPs) [11], and M1-AQP4, able to form tetramers but not OAPs. M1-AQP4 reduces the OAP size when in combination with M23-AQP4 [12]. Using a readthrough mechanism, about 10–20% of AQP4 can be expressed with a longer C-terminus (AQP4ex), which is important to correctly anchor the OAPs to the perivascular side of the glial endfeet and to allow AQP4 phopsphorylation, the function of which is still under investigation [13, 10]. Interestingly, AQP4ex is critical in the triggering event of AQP4 alterations in GBM, and it has been proposed as a potential early biomarker of GBM progression [14].

In studies in which OAPs analysis has been performed by freeze-fracture electron microscopy (FFEM), a correlation between the increase in the grade of malignancy of astrocytomas and the decrease in the amount of OAPs has been reported. The reduction of OAPs is not due to the upregulation of tetrameric M1-AQP4 versus M23-AQP4 expression but rather to the disaggregation of OAPs in tetramers [15, 16].

Using glioma cell lines, we have recently demonstrated that AQP4 tetramer expression potentiates glioma cell invasiveness ability while AQP4-OAP expression drives glioma cells towards the apoptotic path, indicating a key role for AQP4 aggregation state in glioma cell biology [17].

Here, we tested the hypothesis that phenotypic features reported for GBM cells expressing AQP4 tetramers or AQP4-OAPs could be exported, via EVs, to recipient tumour cells and influence their features.

Therefore, in the present study, the size, nature and cargo of the major subclasses of EVs generated by GBM cells, expressing either M1-AQP4 (forming AQP4-tetramers) or M23-AQP4 (forming AQP4-OAPs), have been analysed for their ability to activate the invasiveness or apoptotic pathways of recipient tumour cells.

The results show that EVs generated from GBM cells expressing AQP4-tetramers potentiate the invasiveness ability of recipient cells, while EVs generated from GBM cells expressing AQP4-OAPs favour their apoptotic path, indicating AQP4 as an important cargo in EV mediated communication in glioma.

## Results

### Human GBM cells generate EVs containing AQP4 protein

The possibility that AQP4 protein could be released by glioma cells in a tumour microenvironment was first investigated. We used serum starvation conditions that are recommended for recovery of EVs [18]. In particular, serum-starved cultures of the highly aggressive human glioma cell line U87 either in control conditions (WT) or selectively transfected with the M23-AQP4 isoform (forming AQP4-OAPs and henceforth called AQP4-OAPs) or with the M1-AQP4 isoform (forming AQP4-tetramers and henceforth called AQP4-tetramers) were analysed.

**Fig.1:**
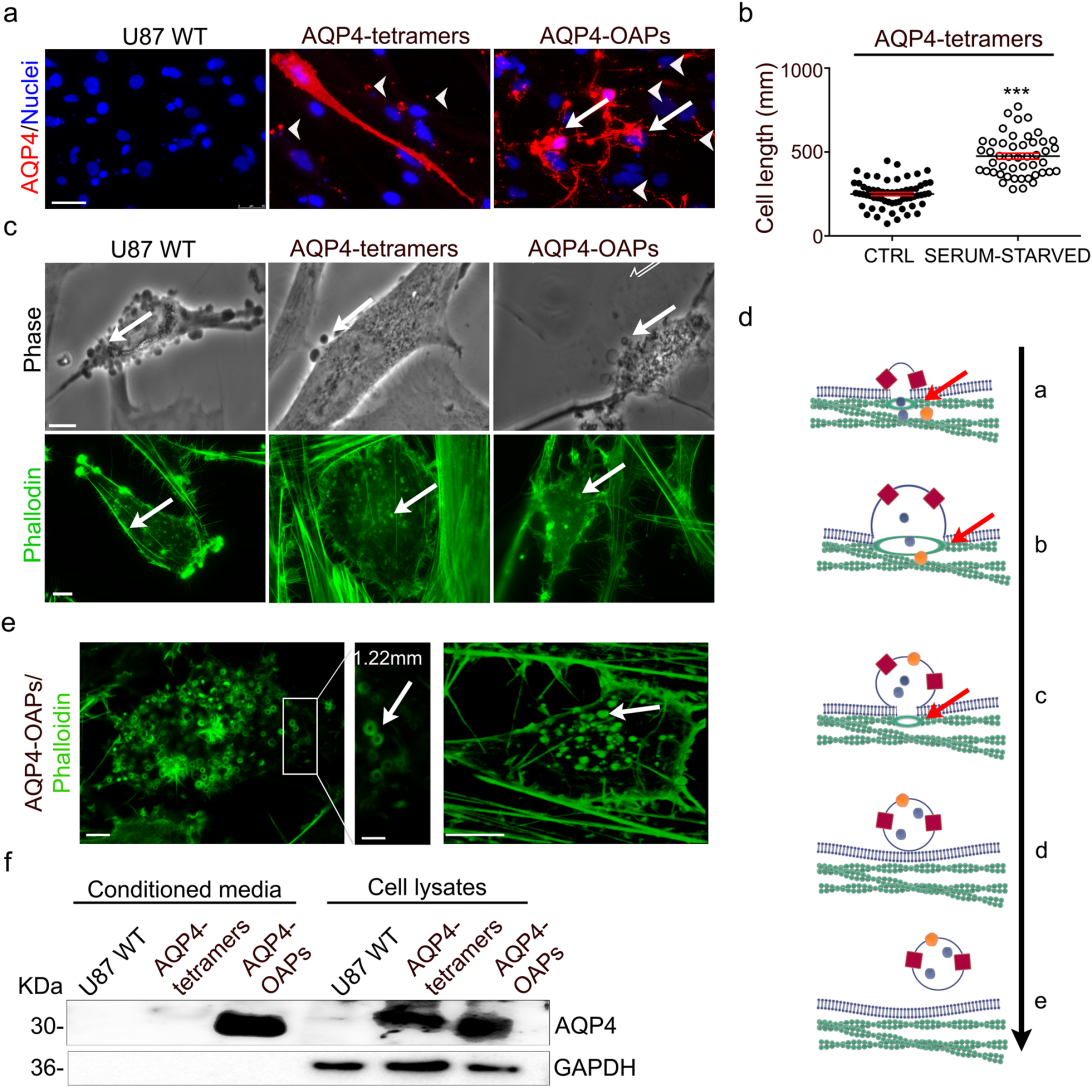
Effect of serum starvation conditions on U87 cell morphology after transfection with AQP4-tetramers or AQP4-OAPs.** a** Epifluorescence images of U87 WT and U87 expressing AQP4-tetramers or AQP4-OAPs cultured in serum-free medium. AQP4 staining is shown in red and DAPI in blue. The arrows indicate cells with altered morphology. The arrowheads indicate the vesicles-like structures positive for AQP4 staining. Scale bar 50 μm. **b** Scatter dot plot showing the quantification analysis of cell length performed for U87 expressing AQP4-tetramers in serum starvation conditions (STARVED) compared to serum containing medium (CTRL). Error bars in the scatter dot plots represent the mean ± SEM of three independent experiments. ***P < 0.0001; 50 fields, n = 4, Student’s t-test. **c** TOP Live phase contrast image of U87 WT and transfected with AQP4-tetramers or AQP4-OAPs isoform. The arrows indicate heterogeneous vesicles at the cell surface. Scale bar 5 μm; *BOTTOM:* Epifluorescence images of U87 WT and transfected with the AQP4-tetramer or the AQP4-OAP isoform stained with 488 labelled-Phalloidin to visualize F-Actin. The arrows indicate the appearance of actin rings**.** Scale bar 10 μm. **d** Drawing/Diagram showing the initiating role of actin ring in delineating the future EVs. A- actin ring formation at membrane budding bottom, B- actin ring expansion increasing the diameter of the neck of growing vesicles, C- actin ring contraction reducing the diameter of the neck of growing vesicles, D- detachment of vesicles from plasma membrane and restoration of actin fibers, E- release of vesicles from donor cell. Spheres represent vesicle cargoes. Red cubes represent AQP4 protein. The arrows indicated the actin rings. **e** LEFT: 3D confocal reconstruction images of U87 expressing AQP4-OAPs stained with 488 labelled-Phalloidin to visualize F-Actin showing the high density of actin-ring structures. Scale bar 5 μm. The boxed area is enlarged and the arrows indicate the ring and the approximate size of their diameters. Scale bar 2 μm; On the RIGHT the arrow showing the appereance of spheric structures fully coated of F-actin. Scale bar 5 μm. **f:** Western blot analysis of AQP4 expression in conditioned media and cell lysates of U87 WT and transfected with the AQP4-tetramers or the AQP4-OAPs isoform as indicated in each lane. Each band represents the monomeric form of the proteins. GAPDH was used as loading control

In line with previously reported experiments performed in normal growth medium [17], AQP4 immunofluorescence images show a profound alteration in cell morphology and cytoskeleton in U87 cells overexpressing AQP4-OAPs compared to control conditions (Fig. 1a). Differently, under starvation conditions, the expression of AQP4-tetramers is also able to induce changes in cell shape with U87 cells acquiring an elongated morphology with a two-fold greater length than in control conditions (475 ± 17,66 and 249.6 ± 10.26 μm, respectively). This suggests that U87 cells overexpressing AQP4-tetramers are more prone to migration [19] (Fig. 1b). Moreover, immunofluorescence images show that both U87 cells overexpressing AQP4-tetramers and U87 cells overexpressing AQP4-OAPs shed extracellular AQP4 positive vesicle-like structures which were investigated in more detail by phase contrast microscopy (Fig. 1c) and F-actin staining (Fig. 1c, bottom and Fig. 1e).

By phase contrast analysis, vesicular structures of heterogeneous size on the surface of the U87 WT cell membrane and U87 selectively transfected with both AQP4 isoforms are distinguishable (Fig. 1c, top). Actin cytoskeleton visualized by fluorescent-labelled Phalloidin shows, in all the three conditions analysed, numerous ring structures corresponding to the neck of budding EVs (Fig. 1c, bottom and Fig. 1d) [20, 21]. In particular, U87 cells expressing AQP4-OAPs show a high density of actin rings (Fig. 1e, left) with diameters of 1.22 ± 0.02 μm. Such diameters are consistent with the size of vesicles. Moreover, 3D confocal reconstruction of OAPs expressing U87 cells shows many F-actin rich regions resembling vesicular structures completely coated in filamentous actin (Fig. 1e, right).

Cell lysates of both cell lines and their conditioned media were separately analysed by western blotting to assess the release of AQP4 containing vesicles by GBM cells.

The results show the presence of AQP4 protein in the GBM cell lysates as well as in the conditioned media. The culture media are void of GAPDH, suggesting that AQP4 extracellular release is not a consequence of contamination by intracellular proteins due to the presence of dead cells (Fig. 1f).

Taken together, the results show that, under starvation conditions, AQP4 expression induces different morphological changes depending on its aggregation state. AQP4-OAP expression induces morphological changes similar to the apoptotic volume decrease (AVD) shape that was already reported for cells grown in regular growth medium [17], whereas the expression of AQP4 tetramers induces a significant cell elongation. More interestingly, starvation induces the formation and release of AQP4 containing vesicles which are particularly enriched in OAPs expressing cells.

### AQP4-OAP expression in GBM cells triggers the release of EVs in the extracellular space through the formation of “beads-on-a-string” apoptopodia

**Fig.2:**
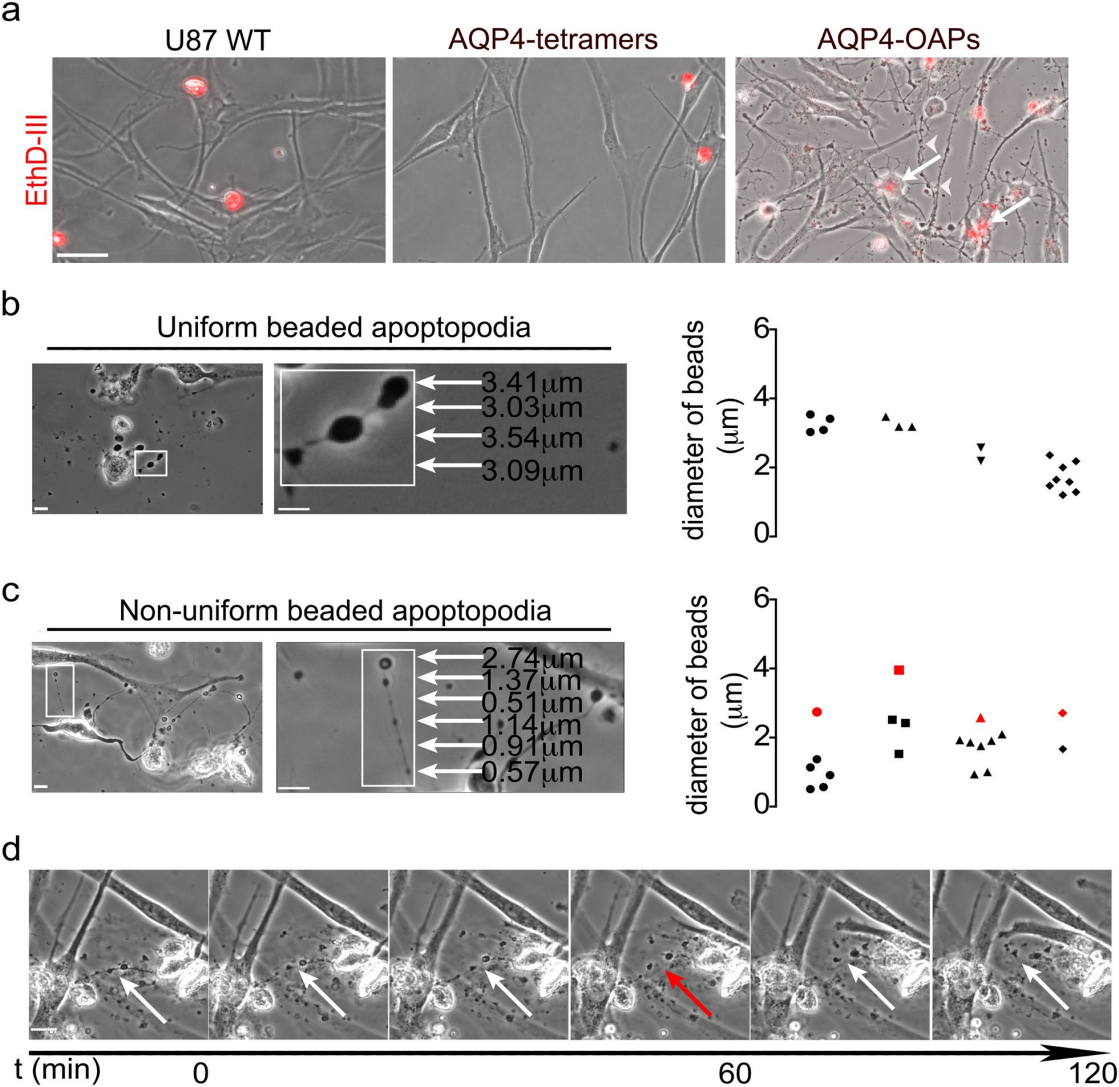
Formation of beaded apoptopodia by U87 cells expressing OAPs. **a** Phase contrast and Ethd-III staining (red) images of U87 WT and U87 expressing AQP4-tetramers or AQP4-OAPs as indicated. The arrows and arrowheads show apoptotic U87 cells expressing OAPs and derived apoptotic EVs, respectively. Scale bar 50 μm. **b** LEFT: Live phase contrast images of U87 expressing OAPs forming uniform beaded apoptopodia. Scale bar 10 μm. The boxed area is enlarged and the arrows indicate beads and relative diameters. Scale bar 5 μm; RIGHT: Quantitation of the diameter of vesicle-like structures on uniform beaded apoptopodia for 4 different fields of three independent experiments. **c** LEFT: Live phase contrast images of U87 expressing OAPs forming non-uniform beaded apoptopodia. Scale bar 10 μm. The boxed area is enlarged, and the arrows indicate beads and relative diameters. Scale bar 5 μm; RIGHT: Quantitation of the diameter of vesicle-like structures on non-uniform beaded apoptopodia for 4 different fields. The red shape indicates the diameter of the largest bead at the tip of each strand of apoptopodia. **d** Time-lapse images monitoring formation of individual vesicles through fragmentation of beaded apoptopodia. A series of time-lapse images taken at 10-min intervals is shown. The white arrows indicate the string. The red arrow indicates the release of vesicles from the string at the 60 min time point. Scale bar 20 μm. (see also Additional file1)

Since the EV secretion is far more pronounced in AQP4-OAPs expressing GBM cells, we analyzed these cells in more detail. We performed a viability test assay using ethidium homodimer (EthD-III) to visualize cells with damaged plasma membrane. The results indicate that the plasma membrane of most cells transfected with AQP4-OAPs, unlike that of WT cells or cells transfected with AQP4 tetramers, is damaged and shows many EVs positive to the staining indicating the presence of DNA content (Fig. 2a).

A more detailed analysis of AQP4-OAPs expressing cells reveals multiple narrow membrane protrusions appearing as typical apoptotic cell structures called “beads-on-a-string” [22], shown in Fig. 2b and Fig. 2c. In particular, two subclasses of beads-on-a-string structures are recognizable, based on whether the ‘beads’ on the apoptopodia are uniform (Fig. 2b) or non-uniform (Fig. 2c) in size. The ‘beads’ found on uniform beaded apoptopodia are predominately 3 μm (2.435 ± 0.1973, n = 17) in diameter, while ‘beads’ found on non-uniform beaded apoptopodia exhibit different patterns in size, with diameters ranging from 0.5 to 4 μm for the largest ‘bead’ at the tip of each strand of apoptopodia. It is worth noting that the diameter of ‘beads’ at the tip of the strand is up to sixfold larger than the other beads on the same string.

After the formation of beaded apoptopodia, the ‘beads’ often fragment and release sections of the apoptopodia or individual vesicles (Fig. 2d). As these vesicles are released by apoptotic cells, they are classified as apoptotic bodies. An additional movie file shows this in more detail (see Additional file 1).

These findings suggest that OAP expression in U87 cells induces the formation of “beads-on-a-string” vesicles released in the extracellular space through fragmentation of beaded apoptopodia.

### AQP4-containing EVs are actively transferred between glioma cells

Based on the observation that human GBM cells may actively generate and secrete EVs of different origins, we next analysed the whole pattern of EVs released in the extracellular space from U87 WT or transfected with AQP4-tetramers or with AQP4-OAPs. For this purpose, conditioned media derived from the above-mentioned three cell lines after 48 h-cultures were subjected to differential ultracentrifugation (DUC) [23]. Pelleted materials recovered at low (300 g), medium (2000×*g* = 2 K), high (10,000×*g* = 10 K) centrifugation speed and ultracentrifugation pellet (100,000×*g* = 100 K) were analysed by western blotting.

In these culture conditions, less than 20% of cell death is generally observed and dead cells are recovered in the 300 g pellet. The EVs pelleting at 2 K likely represent the largest vesicles also containing apoptotic bodies and apoptotic cell fragments, such as vesicles released by apoptopodia or oncosomes (large EVs), whereas EVs pelleting at 10 K likely represent small vesicles (small EVs), and the smallest EVs pelleting at 100 K (micro EVs) also contain a commonly studied subfraction of EVs called exosomes. The diameter analysis shows the highest representation of the largest vesicles in the 2 K pellet, resulting in a mean size of 4.5 m (4.49 ± 0.14 m), whereas vesicles in the 10 K pellet had a mean size below 2 μm (1.67 ± 0.07 μm) and below 1 μm (0.8 ± 0.03 μm) for the 100 K pellet (see Additional file 2).

**Fig.3:**
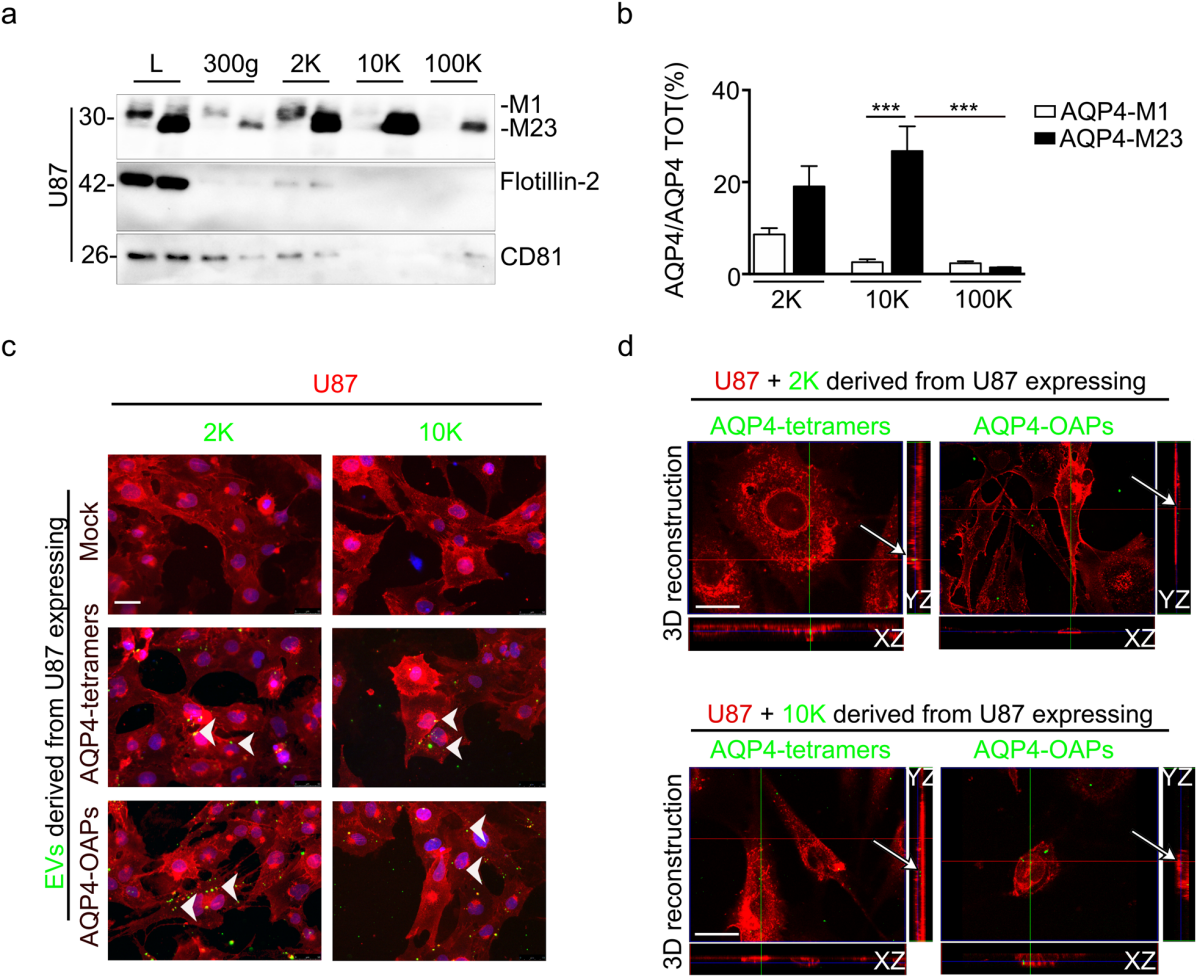
Analysis of AQP4 distribution in EVs released from GBM U87 cells.** a** Immunoblot detection of AQP4 expression levels in cell lysates (L) and pelleting fractions (300 g, 2 K, 10 K and 100 K) derived from conditioned media of U87 transfected with the AQP4-tetramers (M1) or the AQP4-OAPs (M23) isoform as indicated in each lane. Flotillin-2 is used as a marker of large EVs (2 K pellet) and CD81 as an exosomal marker (100 K pellet). **b** Densitometric analysis of the immunoblot in **a** showing AQP4-M1 and AQP4-M23 enrichment in EVs subtypes. Values are expressed as the ratio of AQP4 expression in the fractions /AQP4 total expression (%) ± SEM ***P < 0.0005, n = 3, two-way ANOVA/Tukey’s test. **c** Epifluorescence images of U87 WT used as recipient cells treated with 2 K and 10 K EVs-derived from U87 transfected with empty vector (Mock), AQP4-tetramers or AQP4-OAPs after 24 h of incubation. Recipient cell membrane is stained with WGA (red), while EVs are stained in green for AQP4. DAPI for nuclear staining is in blue. The arrowheads showing perinuclear localization of EVs in recipient cells. Scale bar 50 μm. **d** Single optical intracellular plane of 3D confocal reconstruction and relative YZ and XZ slices showing internalization of large (2 K) and small (10 K) EVs in recipient cells. Recipient cell membranes are stained with WGA (red), AQP4 staining is shown in green. The arrows indicate internalization or interaction of EVs with plasma membrane of recipient cells. Scale bar 20 μm

Western blot results (Fig. 3a) confirm that both AQP4 isoforms M1 and M23 are contained in U87 cell-derived EVs distinct subtypes.

Well-known markers were used to evaluate the cellular origin of the EVs subtypes such as Flotillin-2 and CD81.

Flotillin-2, a scaffolding protein participating in the formation of caveolae or caveolae-like vesicles, is expressed only in the largest vesicle fractions (2 K pellet), indicating the biogenesis mechanism of these subtypes of EVs. CD81, widely used as classical exosome marker [24], is found in 100 K pellets, indicating the exosomal origin of part of these subtypes of EVs.

AQP4 densitometric analysis (Fig. 3b) of western blotting in Fig. 3a demonstrated that a statistically significant difference was found in the amount of AQP4-M1 and AQP4-M23 in the 10 K fraction (2.78 ± 1.4 and 26.76 ± 5.3%, respectively), while no statistical significance exists within the 2 K and 100 K fractions or between them.

Collectively, these results confirm that human GBM cells release a large range of EVs, which are partially separated by their pelleting properties, and demonstrate that both isoforms of AQP4 protein are actively secreted but diversely enriched in the whole pattern of GBM-derived EVs subtypes as well as in exosome fractions.

Then we focused our attention on the EV-mediated communication mechanism between glioma cells. To this task, large EVs and small EVs (derived from the 2 K and 10 K fractions, respectively) constitutively shed by U87 transfected with AQP4-tetramers or AQP4-OAPs or with the empty vector used as a control (Mock) were added to pre-seeded glioma (U87) untransfected recipient cells and analysed by immunofluorescence after 48 h. The results show that EVs are able to reach and interact with recipient cell membranes (Fig. 3c).

Next, we sought to determine whether the large and the small AQP4-containing EVs are completely internalized by recipient cells. To this end, after a 24 h incubation with EVs, plasma membranes of recipient cells were stained with wheat germ agglutinin (WGA) and analysed by confocal microscopy. 3D confocal reconstruction and intracellular confocal plane images of recipient cells and the relative xz- and yz-planes show the presence of large and small EVs within the cell membrane and in the intracellular space. These results indicate that AQP4 protein is transferred between tumour cells in an EV-dependent manner (Fig. 3d).

### Large EVs derived from AQP4-tetramers expressing cells increase metastatic potential in glioblastoma multiforme recipient cells

**Fig.4:**
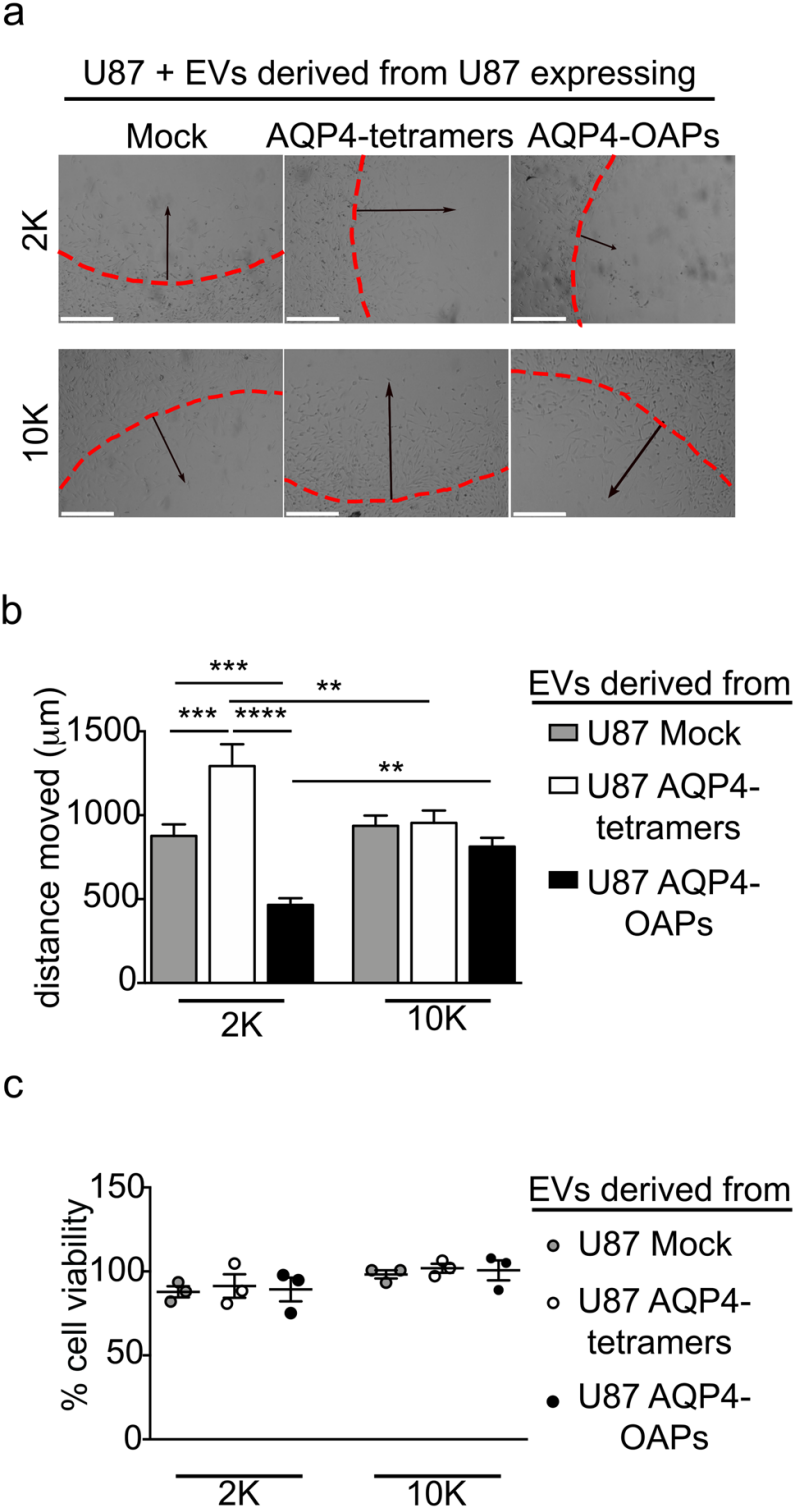
Effects of EVs derived from U87 expressing AQP4-tetramers or AQP4-OAPs on motility and viability of recipient U87 cells. **a** Representative images of U87 recipient cells treated with the 2 K and 10 K fractions of U87 transfected with Mock, AQP4- tetramers or AQP4-OAPs after 48 h migration into an agarose spot containing FBS. Lines denote the edge of the agarose spots. The arrows indicate the direction of chemoinvasion. Scale bar 500 µm. **b** Quantification of the maximum distance from the edge reached by cells reported in **a**, in the spot radius direction. Values are expressed as mean ± SEM ****P < 0.0001, ***P < 0.001, **P < 0.005 and *P < 0.05, n = 4, two-way ANOVA/Tukey’s tests. **c** MTT assay results showing U87 recipient cells viability after 72 h incubation with 2 K and 10 K EVs derived from U87 WT (Mock) and expressing AQP4-tetramers or AQP4-OAPs. The results are represented as a mean ± SEM, n = 3, two-way ANOVA/Sidak’s test

To assess the role of different sized EV in cancer cell–cell communication, we sought to determine whether AQP4 expressing cell-derived EVs affect the invasive response of receiving cells. In particular, we focused on chemotaxis as a hallmark in events ranging from inflammation to cancer progression. The agarose-FBS chemotactic invasion assay [25] was performed as in Fig. 4a. Recipient cells were incubated for 48 h selectively with 2 K and 10 K fractions derived from U87 transfected with Mock, AQP4- tetramers or AQP4-OAPs with the addition of low-serum chemotaxis medium. The maximum distances from the edge of the agarose spot reached by the cells along the radius was measured (Fig. 4b).

Significant effects upon chemotaxis were observed in cells treated with 2 K EVs fraction derived from U87 expressing AQP4-tetramers compared to Mock and U87 expressing AQP4-OAPs. The results show that the distance travelled by cells treated with the 2 K EVs fraction derived from U87 expressing AQP4-tetramers were also significantly higher than their 10 K EVs treated cell counterparts. To control for any effects of the EVs on cellular proliferation influencing the outcome of these assays, a viability assay (MTT) was performed at time points used for invasion assays. There were no significant differences in growth observed with any EV treatment (Fig. 4c).

These data indicate that EVs shed by glioma cells expressing AQP4-tetramers export the pattern of their cells of origin to the receiving cells.

### Large EVs derived from AQP4-OAPs expressing glioma cells increase apoptotic pathway through caspase activation

**Fig.5:**
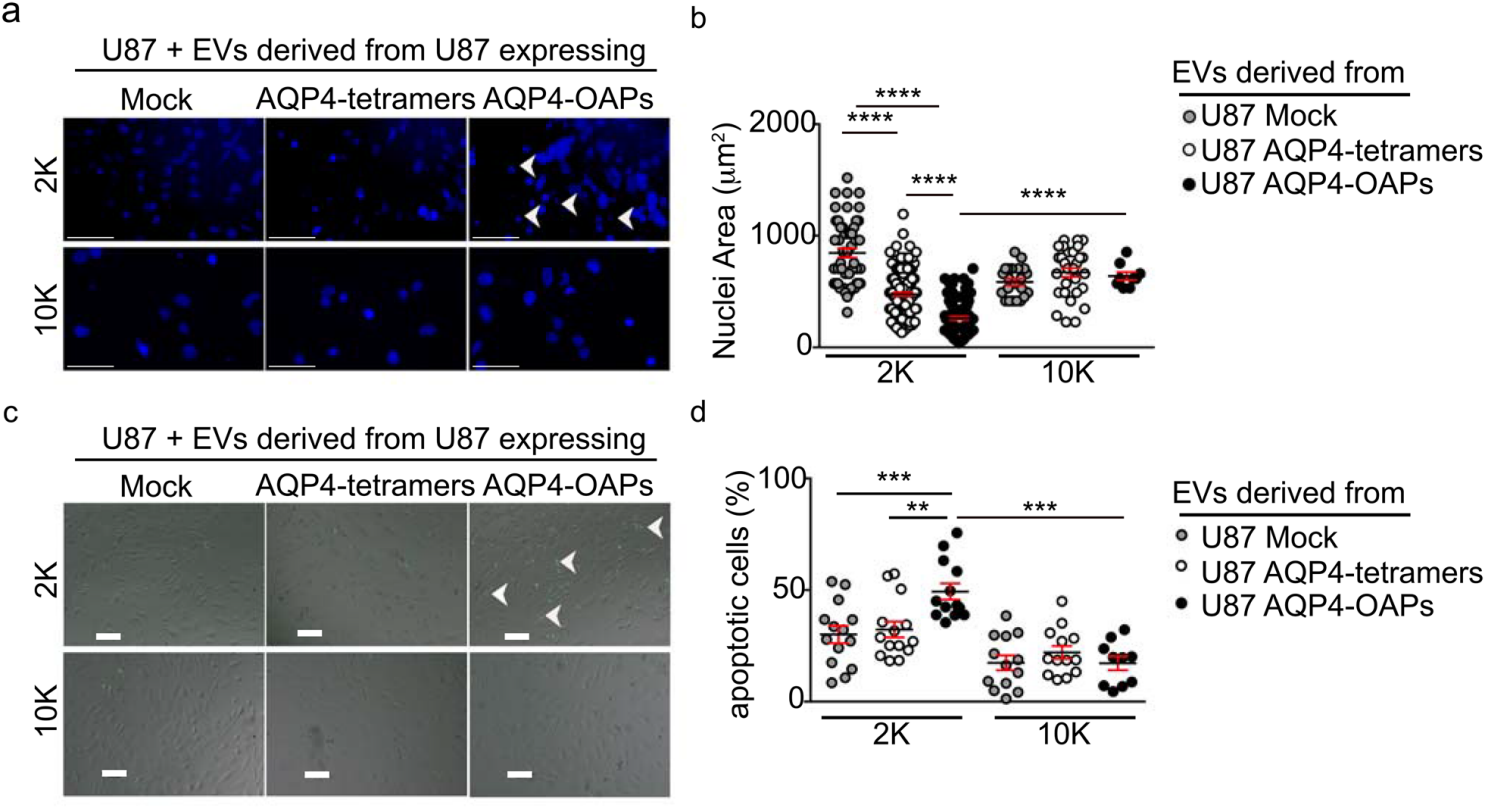
Effects of U87 expressing AQP4-tetramers or AQP4-OAPs-derived EVs on apoptotic activation of recipient U87 cells.** a** Representative image of U87 cells treated with 2 K and 10 K EVs derived from U87 expressing Mock, AQP4-tetramers or AQP4-OAPs as indicated and stained with DAPI to visualize Nuclei after 72 h of incubation. Scale bar 100 µm. **b** Dot plot showing the analysis of the nuclear area of U87 cells treated with 2 K and 10 K EVs derived from U87 expressing Mock, AQP4-tetramers or AQP4-OAPs**.** Values are expressed in µm^2^ and represent mean ± SEM. ****P < 0.0005, n = 3, two-way ANOVA/Tukey’s tests. **c** Epifluorescence images of U87 cells treated with 2 K and 10 K EVs-derived U87 expressing Mock, AQP4-tetramers or AQP4-OAPs after 72 h of incubation. The activity of Caspase-3/7 was detected by CellEvent™Caspase-3/7Green (green) detection reagent. Scale bar 100 µm. **d** Quantification of apoptotic cells in **c**. Results are expressed as the % of the positive cells per field and represent the mean ± SEM. **P < 0.005, ***P < 0.001, ****P < 0.0005, n = 4, two-way ANOVA/Tukey’s test

Despite no significant differences in the recipient cells viability was observed after treatment with any EV subfractions (Fig. 4c), an increase in fragmented and condensed nuclei was found in the recipient cells after exposure to 2 K EVs derived from U87 expressing AQP4-OAPs in comparison to cells exposed to EVs derived from U87 expressing Mock or AQP4-tetramers (Fig. 5a) and to their counterparts exposed to 10 K EVs.

In particular, the analysis shows a statistically significant reduction in the nuclei area for cells treated with 2 K EVs derived from U87 expressing AQP4-OAPs compared to cells treated with either 2 K or 10 K EV fractions derived from all the above-mentioned cell lines (2 K derived from U87 Mock: 946 ± 50, AQP4-tetramers: 460 ± 19, AQP4-OAPs: 261 ± 14; 10 K derived from U87 Mock: 561 ± 29, AQP4-tetramers: 723 ± 43, AQP4-OAPs 816 ± 70) (Fig. 5b).

Then U87 cells after incubation with EVs derived from U87 selectively expressing Mock, AQP4-tetramers or AQP4-OAPs were analysed for caspase 3/7 activation.

As expected, the exposure to 2 K EVs derived from U87 expressing AQP4-OAPs leads to an increase in the number of receiving cells labelled for activated caspase 3/7 compared to the exposure to EVs derived from U87 expressing Mock or AQP4-tetramers (Fig. 5c). The analysis shows an increase in the percentage of apoptotic cells in the population of cells treated with 2 K EVs derived from U87 expressing AQP4-OAPs compared to cells treated with either 2 K or 10 K EV fractions derived from all the above-mentioned cell lines (2 K derived from U87: Mock: 30.09 ± 3.8, AQP4-tetramers: 32.32 ± 3.5, AQP4-OAPs: 49.33 ± 3.6; 10 K derived from U87 Mock: 17.37 ± 3.3, AQP4-tetramers: 22.05 ± 2.9, AQP4-OAPs 17.18 ± 3) (Fig. 5d).

These data indicate that EVs shed by glioma cells expressing AQP4-OAPs export the pattern of their cells of origin to surrounding cells.

## Discussion

Multiple populations of glioblastoma (GBM) cells coexist within a single tumour and communicate by a variety of extracellular signals increasing the complexity of the disease, thus suggesting a potential significance in understanding how signals produced by a population of glioma cells affect the surrounding tumour cells response.

In the context of GBM, EVs have been shown to be able to affect phenotypes of stromal counterpart cells. EVs derived from GBM cells have been implicated in endothelial cell (EC) proliferation, migration and tubulogenesis via delivery of angiogenic proteins and RNA to microvascular ECs [26]. Furthermore, the crosstalk between GBM and astrocytes via EVs is critical in the evasion of tumour cell apoptosis, contributing to GBM aggressiveness and proliferation [27].

In the present study, we demonstrate that glioma cells expressing AQP4 can export their metastatic or apoptotic phenotypes toward tumour surrounding cells, and this phenomenon is, at least in part, mediated by intercellular transfer of EVs.

The interest in acquaporins (AQPs) trafficking is justified by the role demonstrated for AQPs in brain tumour pathogenesis. In particular, aquaporin-1 (AQP1) is important in tumour growth and spread [28] and AQP4 protein has a crucial role in vasogenic oedema that increases the mortality related to brain tumours [29].

Moreover, in the last five years, in five different studies, AQP4 has been found in association with EVs in CNS tissues in Alzheimer’s Disease (AD), stress-induced exhaustion disorder (SED), traumatic brain injury (reviewed here [30]), suggesting the growing interest in these fields.

These studies, based on data from CSF and blood samples, demonstrated the increase of AQP4-containing EVs release in pathological conditions in diseased patients and animal models (www.microvesicle.org). However, none of these studies investigated in detail the AQP4 aggregation state in EVs.

Besides the role in brain edema, we have previously demonstrated that the aggregation state of AQP4, ranging from tetramers to different sized OAPs, can influence glioma cell fate as follows: AQP4-OAP expression leads to cell shrinkage with alteration in the actin cytoskeleton and apoptotic outcome being therefore "deleterious" for glioma cell survival, while AQP4-tetramer expression increases glioma invasive capability being therefore "beneficial" for glioma cells [17]. This finds its basis in the functional role amply reported for M1-AQP4 in favoring cell migration both in healthy astrocytes and in glioma cells [31, 32].

Furthermore, considering the reduced amount of OAPs found in the human GBM sample, we have previously speculated that this could be considered a survival strategy adopted by the glioma cells that exert the decrease in OAPs, through disaggregation of OAPs in tetramers, to escape apoptosis and to increase the grade of malignancy [33].

The different phenotypic features activated by AQP4 aggregation/disaggregation state in glioma cells prompted us to hypothesize that different signal cascades could be transferred, via EVs, to surrounding tumour cells from metastatic or apoptotic glioma cells expressing AQP4-tetramers or AQP4-OAPs, respectively.

The study has been conducted using the most widespread experimental model of glioma: the U87 cell line as recipient cells and U87 cells transfected with AQP4–tetramers or AQP4-OAPs as donor cells, in serum withdrawal conditions. It is well known that tumour cells undergoing serum starvation in vitro try to adapt to the modified environment supporting the tumour growth [34]. Moreover, it is also likely that cells having acquired constitutive tolerance for nutrient and oxygen deficiency show an increase in malignancy [35]. First, we obtained direct evidence that AQP4 aggregation states trigger different morphological changes, also under starving conditions. If AQP4-OAP expression induced the AVD like shape [17], the expression of AQP4 tetramers led to a significant cell elongation.

Given the well-known ability of glioma cancer cells to generate EVs [24, 23], we hypothesize that morphological adaptation of U87 expressing AQP4–tetramers is predictor of the EV secretion that is facilitated by the greater cell plasma membrane surface and by more endosomal machinery available in the larger cells.

The advanced apoptotic phenotype of U87 cells expressing AQP4-OAPs is a predictor of EV release, given that apoptotic cells release more EVs than viable cells. Moreover, we found that OAP expression in U87 cells induces the formation of “beads-on-a-string” vesicles released in the extracellular space through fragmentation of beaded apoptopodia. These membrane protrusions, peculiar of apoptotic cells, have recently been reported for other cell lines such as apoptotic monocytes [22]. Since tumour cells acquire tolerance for nutrients and oxygen deficiency and increasing malignancy, it is not surprising that U87 cells also exhibit features, namely the enhanced ability to grow under serum-starved conditions and altered cell shapes [36].

The U87 cell line is well known to be prone to produce a large and heterogeneous population of EVs also without any treatment and, at the same time, to uptake autologous EVs [37, 23].

To date, the studies available on EVs derived from untransfected U87 cells reveal that EVs are capable of conferring on normal counterpart cells the transformed characteristics of cancer cells; promoting radiation resistance; and increasing proliferation and invasion [38, 38] (reviewed in [40]).

The ability of glioma cells to generate EVs is also sustained by the presence of actin rings at the membrane level that facilitate membrane blebbing either in exocytosis or endocytosis processes [41]. The physical dynamics that fold sub-regions of the plasma membrane into vesicles involves modulation of the actin cytoskeleton playing a key role in the formation and the release of EVs [42].

The actin rings form the neck of growing EVs when they are still in contact with the plasma membrane, next contributing to reducing the diameter of the neck of budding vesicles before being shed from the plasma membrane. Therefore, the presence of actin rings is predictive of continuity between the plasma membrane and forming EVs such as in endocytosis [21, 20].

From the analysis of actin cytoskeleton we demonstrate that glioma cells expressing AQP4-OAPs show a higher density of actin rings and many F-actin-rich regions resembling vesicular structures completely coated in filamentous actin. This is in line with previous experiments that showed that glioma cells expressing OAPs display a higher content of F-actin, in turn compatible with their very low migration potential being directed to apoptosis rather than invasiveness [17].

The preliminary analysis of protein released in extracellular space by glioma cells, either expressing AQP4-OAPs or AQP4–tetramers, confirms the presence of AQP4, suggesting that, apart from its role in cell physiology, AQP4 also exists as a secreted protein. Although it has been postulated that brain cancer cells distribute aquaporins (AQPs) between cells via EVs [43, 44], as occurs in the kidney where AQP1 and aquaporin-2 (AQP2) have been found in urinary exosomes, the presence of an AQP, namely AQP4, in EV cargoes derived from glioma cells has been reported here for the first time.

Based on the evidence that EVs are released from all cells in varying sizes and with different contents, we isolated them by exploiting their pelletting properties.

Glioma-secreted EVs mainly appear to be of plasma membrane/ ‘shed-vesicle’ origin and belonging to three distinct subpopulations. Despite the numerous studies, the nomenclature and the boundaries between subpopulations of EVs are still under debate. Here we have focused on two (large and small) subpopulations of EVs, because of their abundance compared to the smallest vesicle fraction and their genesis from plasma membrane where AQP4 protein resides.

Our findings indicate that both AQP4-M1 and AQP4-M23 proteins are readily detected in both subpopulations. In detail, AQP4-M1 and AQP4-M23 contents are comparable in large EVs, while M23 protein has higher levels in small EVs. Interestingly, we found a reduced amount of AQP4 expression levels in the cell lysate of U87 expressing M1 compared to U87 expressing M23 protein. This could be ascribed to the sub-optimal translation initiation signal for the M1 start codon [45, 46] and to perturbations in the translation initiation mechanism that occur in cancer cells in stress conditions such as serum withdrawal [47, 48].

Several studies in the past decades have shown that large and small EVs, by transferring several bioactive molecules, affect the phenotypic features of receiver cells, increasing their migratory capability, proliferation and therapy resistance [39].

Finally, in line with this, we demonstrated that EVs derived from U87 expressing AQP4-tetramers or from AQP4-OAPs are able to reach receiving cells, exporting to them the pattern of their cells of origin.

In detail, large EVs derived from more invading glioma cells expressing AQP4-tetramers potentiate the migratory response of receiving glioma cells, while EVs shed by apoptotic glioma cells expressing AQP4-OAPs favour the apoptotic path of receiving cells.

Notably, these large vesicles are more than twofold the size of small and micro vesicles, therefore they can virtually include a larger number of tumor-derived molecules, with a distinct impact on the recipient cells.

It is reasonable that U87 aggressive cells may preferentially load some proteins in large vesicles more than in small EVs to improve cell-to-cell messages aimed to cancer progression.

Intriguingly, large vesicles, such as oncosome, have recently been shown to be enriched in a set of several enzymes involved in cancer cell metabolism and cell cycle and are able to transfer both adhesion and invasion properties from aggressive cell line to the less aggressive counterpart. Moreover, large vesicles have been shown to contain miRNA, mRNA and DNA and genetic aberrations belonging to the cell of origin including copy number variations of genes frequently altered in aggressive tumour cells.(reviewed in [49]).

Since GBM cells exert the redistribution of OAPs in favour of tetramers, the mainly transferred phenotypic trait is invasiveness. Moreover, the apoptotic activation of surrounding cells could be addressed toward less malignant cells or stromal cells.

Therefore, AQP4 EV-mediated transfer could be a tumour-supporting mechanism by which glioma cells can export their tumour-enhancing- phenotype or can promote a phenotypic switch either between the tumour and less malignant tumour cells or among tumour cells and stroma. Also in this latter case, the mirroring of glioma cell traits is useful for tumour propagation.

Therefore, in trying to understand the patho-physiology of glioma, the general increase in AQP4 expression and the redistribution of OAPs in favour of tetramers is useful for tumour propagation as they affect both glioma cells expressing AQP4 and tumour or normal surrounding cells with which they communicate. As it is well known that the impact of EVs may not be often fully caused by any single molecule, one possibility is that EVs contain multiple proteins including AQP4 and other components such as various miRNAs [50] with overlapping functional roles acting in a concerted mechanism to affect the phenotype of recipient cells.

A comprehensive proteome profiling of glioblastoma-derived extracellular vesicles derived from six GBM, including U87, cells lines revealed that levels of 14 EV proteins significantly correlated with cell invasion. Gene levels corresponding to invasion-related EV proteins showed that five genes (annexin A1, actin-related protein 3, integrin-β1, insulin-like growth factor 2 receptor and programmed cell death 6-interacting protein) were significantly higher in GBM tumours [38].

Further studies are needed to investigate the change in gene levels and invasion-related proteins in EVs from GBM cells expressing AQP4-tetramers compared to expressing AQP4-OAPs.

In conclusion, this study demonstrates that invasiveness or apoptosis traits of glioma cells expressing AQP4 protein affect the signal transferred to surrounding cells. By EV-mediated crosstalk, the phenotypic features of donor cells are exported to receiving glioma cells, amplifying the role of AQP4 in glioma cells also in surrounding cells. In terms of the biology of glioma, EV-mediated transfer of AQP4 to surrounding cells acts as a tumour-supporting mechanism, emphasizing the role of AQP4 as a determinant of cell fate and confirming that the redistribution of OAPs in favour of tetramers is useful in propagating tumours and in spreading malignancy.

Thus, it is conceivable that the phenotype previously described as being dependent on AQP4 membrane expression (17) could also be generated by the AQP4 circulating fraction.

## Conclusion

We believe that this study adds knowledge on the complex role of AQP4, different from its well-known primary function of the plasma membrane water channel in tumour biology and in the pathophysiology of glioma providing information on regulating the EV-mediated pro-tumorigenic response.

Moreover, due to the potential use of EVs as a source of both diagnostic and prognostic biomarkers in cancer, many efforts are nowadays focusing on the characterization of EVs specific cargos, in order to select new molecular markers that could help determine specific GBM tumour molecular signatures.

In this view, these results add a piece of knowledge on GBM-derived EVs profile in the function of the state of primary tumours. Identifying AQP4 protein in the composition of GBM-derived EVs can reflect apoptotic or aggressive molecular signatures of primary tumour indicative of aggressiveness or response to chemotherapy.

The presented data motivate future studies exploring AQP4-containing EVs utility in non-invasive diagnosis and monitoring of brain tumour patients.

## Methods

### Cell lines

The cell line U87 MG (ATCC HTB-14), derived from a malignant glioma from a female patient by explant technique [51], was acquired from the ATCC (www.lgcstandards-atcc.org).

Cells were used from passages 174 to 185. Mycoplasma testing was routinely conducted with MycoAlert Substrate (www.bioscience.lonza.com) or by fluorescence staining with DAPI. Cells were cultured in DMEM-F12 (1:1) supplemented with 10% FBS, 100 U/mL penicillin and 100 mg/mL streptomycin, and maintained at 37 °C in a 5% CO_2_ incubator. FBS was omitted in experiments aimed at harvesting EVs due to the presence of endogenous EVs in the FBS itself [52].

### Constructs and transfection

Human M1M23I-AQP4 (also called AQP4-tetramers) and M23-AQP4 (also called AQP4-OAPs) coding sequences were cloned into pTarget (A1410, www.Promega.com) vectors. The previously characterized mutated form of M1-AQP4 (M23I), demonstrated to give rise exclusively to AQP4-tetramers, was used [45].

Twenty-four hours before transfection, cells at 70% confluence were plated using antibiotic-free medium. Transient transfection was carried out using Lipofectamine 3000 (L3000015, www.thermofisher.com) in OptiMEM growth medium according to the manufacturer's protocol. Twenty-four hours later, transfection medium was replaced with serum-free medium to eliminate medium-derived EVs. After 48 h, conditioned medium was collected and analysed for EVs.

### Antibodies

The following primary antibodies were used: rabbit polyclonal anti-AQP4 (H-80) (Santa Cruz Biotechnology Cat# sc-20812, RRID:AB_2274338) diluted 1:400 for immunofluorescence and 1:500 for immunoblot analysis, mouse monoclonal anti-CD81 (Santa Cruz Biotechnology Cat# sc-166029, RRID:AB_2275892) diluted 1:100 for immunoblot analysis, mouse monoclonal anti-Flotillin-2 (Santa Cruz Biotechnology Cat# sc-48398, RRID:AB_627615) diluted 1:200 for immunoblot analysis and mouse monoclonal anti-GAPDH (Millipore Cat# MAB374, RRID:AB_2107445) diluted 1:2000 for immunoblot analysis.

488-labelled Phalloidin (A12379, www.thermofisher.com) was used to stain F-Actin.

EthD-III was used to stain apoptotic nuclei (30,017, www.biotium.com). DAPI was used to stain nuclei (D9542, Merck). WGA staining (W849, www.thermofisher.com) was used to stain cell membrane.

The secondary antibodies used were: donkey anti-rabbit Alexa Fluor 488- (Molecular Probes Cat# A-21206, RRID:AB_2535792) and 594-conjugated (Molecular Probes Cat# A-21207, RRID:AB_141637) for immunofluorescence analysis; goat anti-mouse IgG (H + L) HRP conjugate (Bio-Rad Cat# 170–6516, RRID:AB_11125547), goat anti-rabbit IgG-HRP (Santa Cruz Biotechnology Cat# sc-2004, RRID:AB_631746) for western blotting analysis.

### Immunofluorescence

Cells were fixed in 4% paraformaldehyde for 15 min, washed 3 times in PBS, and permeabilized with 0.1% Triton X-100. After blocking using 2% bovine serum albumin (BSA) for 15 min at room temperature, cells were incubated for 1 h with primary antibodies and washed with PBS/BSA. Cells were finally incubated with Alexa Fluor–conjugated secondary antibodies and mounted with a medium containing 50% glycerol, 1% DABCO in PBS, and DAPI for nuclear staining.

### Live-cell imaging

For all live imaging experiments, cells were seeded in confocal dishes with a glass bottom and were subjected to transfection as described previously. For EthD-III staining, cells were incubated in binding buffer for 10 min with 5 μL of EthD-III according to the manufacturer's protocol 24 h after transfection, washed and analysed. The phase contrast images, epifuorescence and time-lapse were acquired using the BioStation IM-Q device, an incubator equipped with a microscope and a high-sensitivity cooled CCD camera. The acquisition conditions were the following: 20x, 40 × and 80 × magnification, 488- and 594-filter for excitation in Fluorobrite DMEM medium (A1896701, www.thermofisher.com). Images were acquired every 10 min for at least 2 h.

### Epifluorescence and confocal microscopy

Fluorescence labelled cells and vesicles were observed with a photomicroscope equipped for epifluorescence and 16x, 40 × oil PL FL FLUOTAR objective, using the appropriate filter. Digital images were obtained with a DMX1200 camera (Nikon, Tokyo, Japan) and processed using LAS AF software (Leica Application Suite X, RRID:SCR_013673). Once captured, the auto contrast function was applied to all the images using Photoshop CS5 (Adobe Photoshop, RRID:SCR_014199).

All confocal images were obtained with a Leica TCS SP5 and were collected using the 594 and 488 laser lines for excitation and a pinhole diameter of 1 Airy unit. The optical series covered at least 50 optical slices, from the top to the bottom of the cells, with a raster size of 1024*1024 in the x–y planes and a z-step of 0.15 μm between optical slices.

### Isolation of EVs

EVs were isolated by differential ultracentrifugation. Briefly, conditioned medium was centrifuged at 300×*g* for 10 min at 4 °C to pellet floating cells and debris. Supernatant was centrifuged at 2000×*g* for 20 min at 4 °C (2 K pellet), transferred to new tubes, and centrifuged in a fixed angle rotor for 40 min at 10,000×*g* at 4 °C, and finally for 90 min at 100,000 × g in a 70Ti rotor (Beckman, www.beckmancoulter.com) always at 4 °C. All pellets were washed in 5–6 mL of PBS and recentrifuged at the same speed before being resuspended in 200 μL of sterile PBS for labelling or medium for uptake assay. Cells recovered from the first 300 × g pellet were pooled with cells detached from the plates by incubation at 4 °C in PBS-EDTA (DCs) or in trypsin–EDTA (adherent cells) (Gibco, www.thermofisher.com) and counted using a Countess Automated cell counter (Life Technologies Countess Automated Cell Counter, RRID:SCR_020236). Viability was assessed by Trypan Blue stain 0.4% (T10282, www.thermofisher.com) exclusion.

### DiO cell_labelling

After the washing step, each EV pellet was labelled with Vybrant® DiO cell-labelling diluted 1:1000 in serum free-medium ( V22886, www.thermofisher.com) for 20 min at 37 °C, then washed three times for 10 min each at 37 °C with serum-free medium. After centrifugation, each pellet was resupended in 200 μL of PBS and analysed with an epifluorescence microscope for size analysis or used in uptake assay.

### EV size analysis and quantification

20 μL of labelled EV suspensions was mixed with glycerol-based mounting medium, seeded on a slide and immediately visualized with a photomicroscope equipped for epifluorescence at 40 × magnification. The diameters of each EV in every field were analysed using the size measure plugin of Fiji software (Fiji, RRID:SCR_002285). Quantitative analysis was conducted on 5 different fields from each of 3 independent experiments. The results were analysed using GraphPad Prism 6 (GraphPad Prism, RRID:SCR_002798).

### Uptake assay

Twenty-four hours before being assayed, U87 cells, here used as recipient cells, at 50% confluence were plated in 12 or 24 multiwell format. EV suspensions at a concentration of 50 μg/mL were added to recipient cells in a total volume of 1 mL of medium and incubated for 24 h. Later, cells were stained with Wheat Germ Agglutinin (WGA, 1:300 in PBS) for 15 min to highlight the plasma membrane and then subjected to immunofluorescence as detailed above.

### SDS-PAGE

A confluent layer of transfected U87 cells, conditioned medium and derived-EV pellets were washed once with ice-cold PBS and lysed into seven volumes of Lysis buffer (25 mM Tris–HCl, pH 7.4, 100 mM NaCl, 1% NP-40), then lysed on ice for 1 h, and the samples were then centrifuged at 22,000x*g* for 30 min at 4 °C. The supernatants were collected, and the total protein content was calculated using the BCA Protein Assay Kit (71,285-M, www.thermofisher.com). Ten micrograms of protein samples were mixed with 2X Laemmli Sample Buffer (1,610,737, www.Bio-Rad.com) added with 50 mM dithiothreitol, heated to 37 °C for 10 min, resolved in a 13% polyacrylamide gel, and transferred onto PVDF membranes (IPVH00010, www.merckmillipore) for immunoblot analysis.

### Western blotting and densitometric analysis

After transfer, the membranes containing the blotted proteins were blocked and incubated with primary antibodies diluted as described in the Antibodies section. After washings, the membranes were incubated with peroxidase-conjugated secondary antibodies and washed again. Reactive proteins were revealed with an enhanced chemiluminescent detection system (1,705,060**,** www.Bio-Rad.com) and visualized on a Chemi-Doc imaging system (www.Bio-Rad.com). Images were recorded and data analysed with Image lab software (www.Bio-Rad.com).

The Optical density value was determined for equal sized boxes drawn around antibody-stained bands and analysed using GraphPad Prism 6 (GraphPad Prism, RRID:SCR_002798).

### Chemotactic invasion assay

This assay was performed mainly following the Wiggins’sprotocol [53]. Briefly, 0.05 g of low melting point agarose (16,520,100, www.thermofisher.com) was diluted with 10 mL PBS to obtain a solution of 0.5% agarose. It was heated up till boiling point and shaken to reach the complete dissolution. 90 μl of melted agarose was dropped into a 1.5 mL tube, supplied with 10 μL of FBS (AGAR + FBS), as chemoactrant enhancer. 10 μL of agarose-FBS solution was pipetted onto two 12 mm diameter coverslips coated with poly-L-lysine and placed in a 24 multiwell format. After that, the MWs were left for 30 min for the AGAR to cool and for the right spot texture.

20,000 cells in 10% FBS cell culture medium were plated into 24 multiwells and incubated at 37 °C to allow the cells to adhere. After 12 h, the culture media was replaced with 0.1% FBS, containing EVs and the MW was returned to the 37 °C incubator. The purpose of the media change is to ensure no cell proliferation during the experiment. After 48 h, the agarose spots were analysed by measuring the distance moved from the edge toward the center of the spot using the image analysis software ImageJ/Fiji (Fiji, RRID:SCR_002285). The values reported herein are the average of at least three independent experiments, 12 fields of view per treatment, and the error bars represent standard error of mean.

### MTT assay

The effect of EVs pellets on U87 cells viability was assessed using the MTT assay. 8000 cells were plated into MW96 and incubated at 37 °C to allow the cells to adhere. After 12 h, the culture media was replaced with 150 μL of serum-free medium containing EVs and the MW was returned to the 37 °C incubator. After 48 h, 10 μL of tetrazolium MTT (5 mg/mL) (3-(4, 5-dimethylthiazolyl-2)-2, 5 diphenyltetrazolium bromide) was added and the cells were incubated at 37 °C for 4 h. During the reaction, the yellow tetrazolium salt MTT is converted to purple formazan crystals by intracellular reducing equivalents produced by metabolically active cells. Subsequently, 100 μL of acidic isopropanol (0.01 N HCl in isopropanol) was added to each well and mixed thoroughly to dissolve the generated formazan crystals. The spectrometric absorbance value of the wells was read at 595 nm and 620 nm using a microplate reader (www.Bio-Rad.com). Cell viability upon different EV pellets was expressed as the percentage of control cells of 3 readings of three independent EV preparations.

### Nuclear stainining and Caspase-3/7 activity assay 

U87 cells (3 × 10^3^ cells/well) were seeded in a 96-well plate and incubated with EVs for 72 h. After incubation, cells were labelled with 10 μg/mL of DAPI (30 min at 37 °C) for nuclear staining or 1μL of CellEvent™Caspase-3/7Green detection reagent (C10423, www.thermofisher.com) in 100 μL of FBS depleted medium for 30 min at 37 °C in the dark for detection of the activity of Caspase-3/7 according to the manufacturer’s instructions.

Stained cells were observed under an inverted fluorescence microscope. The values reported herein are the percentage of labelled cells/total cells per field of at least three independent experiments, 12 fields of view per treatment, and the error bars represent standard error of mean. Nuclei from 3 fields of at least three independent experiments were automatically detected and the mean nuclear area was calculated with Fiji (Fiji, RRID:SCR_002285).

### Experimental design and statistical analysis

All data represent at least three replicates from independently prepared samples as indicated in the figure legends. Statistical analyses were conducted using GraphPad Prism 6 software (GraphPad Prism, RRID:SCR_002798). All data are reported as the mean ± SEM.

Statistically significant differences were computed using the Student's t test for unpaired data and one-way or two-way Anova with Tukey's multiple comparisons test for multiple statistical comparisons between groups. The significance level was set at *p* < 0.05.

## Supplementary Information

**Additional file 1: **Time-lapse images of U87 expressing AQP4-OAPs monitoring formation of individual vesicles through fragmentation of beaded apoptopodia. A series of time-lapse images is shown at 20× magnification and taken at 10-min intervals from 0 to 120min. The breakdown of the string and vesicles release occurs at the 60 min time point.

**Additional file 2: **Characterization of EVs released from GBM U87 cells.** a: **Scheme of EV isolation by differential ultracentrifugation (DUC) from conditioned medium of human U87 WT cells and related representative images of EVs recovered in each pellet and labelled with vibrant-DIO labelling solution (green). Scale bar 50 μm. **b. **Scatter dot plot showing the distribution of EV diameters in **a **from three independent EV preparations. Error bars in the scatter dot plots represent the mean ± SEM. Note that the difference in size between the three groups is statistically significant.****P < 0.0001; n=30, one-way ANOVA, Tukey’s test. **c: **Epifluorescence images of 2K and 10K EVs-derived U87 WT labelled with lipid-associating fluorescent dye (green) showing perinuclear localization of EVs in recipient cells. Nuclei are stained blue (DAPI). Scale bar 10 μm. **d: **Time-lapse images monitoring kinetic uptake of 10K EVs in recipient cells. Two time-lapse images taken at 1 and 24h after incubation are shown. The arrows indicate the maximum uptake of EVs after 24h. **e**: Single optical intracellular plane showing 3D confocal reconstruction of internalization of EVs in recipient cells. Recipient cell zzzmembrane is stained with WGA (red) and EVs are labelled with lipid-associating fluorescent dye (green). Scale bar 10 μm.

**Additional file 3: **Graphical abstract Extracellular vesicles export phenotypic features of donor cells. Due to their close relation with their cells of origin, EVs derived from the invading glioma cells expressing AQP4-tetramers confer invasive ability on recipient glioma cells. On the contrary, EVs derived from apoptotic glioma cells expressing AQP4-OAPs transfer apoptotic traits to recipient glioma cells. In view of the upregulation of the AQP4-tetramers compared to the assembly state of OAPs, EVs transfer could be a tumour-supporting mechanism by which glioma cells can export their tumour-enhancing phenotype.

## Data Availability

All data generated or analyzed during this study are included in this published article.

## Abbreviations

AQP4: Aquaporin-4
OAPs: Orthogonal arrays of particles
FFEM: Freeze-fracture electron microscopy
AQP1: Aquaporin-1
GBM: Glioblastoma multiforme
WT: Wild type
EthD: Ethidiumhomodimer III
AQP0: Aquaporin-0
AVD: Apoptotic volume decrease
EVs: Extracellular vesicles
TME: Tumour microenvironment
GAPDH: Glyceraldehyde-3-Phosphate Dehydrogenase
DUC: Differential ultracentrifugation
WGA: Wheat germ agglutinin
300 g: (300xg)
2 K: (2.000xg)
10 K: (10.000xg)
100 K: (100.000xg)
EC: Endothelial cells
